# Supplementary material for: C. elegans is not a robust model organism for the magnetic sense
Source: Commun Biol. 2023 Mar 4;6:242. doi: 10.1038/s42003-023-04586-8 (PMC9985618; doi:10.1038/s42003-023-04586-8)
Supplement: Supplementary file 2 — Supplementary information [file 42003_2023_4586_MOESM2_ESM.pdf]

Supplementary Information

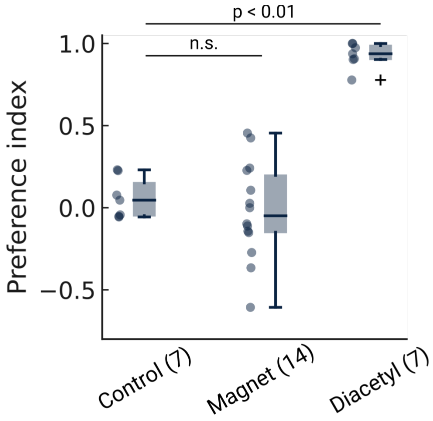

Supplementary Figure 1. Strong neodymium magnets do not elicit magnetotaxis in an ancestral strain of *C. elegans* N2 animals. Preference index of the animals after exposure to either a non-magnetic metal (Control), a neodymium metal (Magnet) or a known attractant (Diacetyl). The boxplots follow Tukey's rule where the middle line indicates the median, the box denotes the first and third quartiles, and the whiskers show the 1.5 interquartile range above and below the box. Significance was assessed using a Mann-Whitney U-test. Numbers in parentheses denote the number of independent replicates (n = biologically independent experimental plates).

.

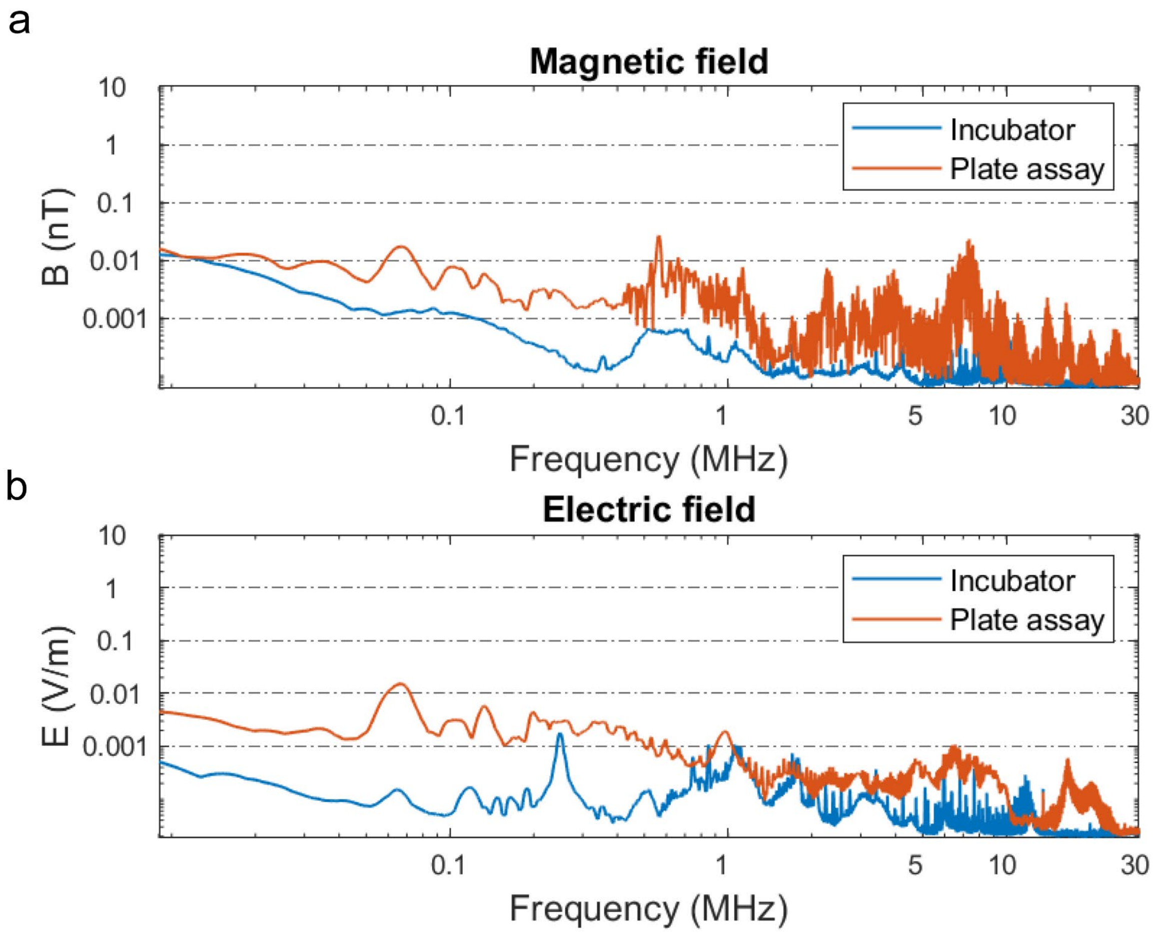

Supplementary Figure 2. Measurements of time-dependent electromagnetic fields. Graphs showing the (a) magnetic and (b) electric components of electromagnetic fields present in the plate assay room (orange) and the incubator (blue). between 0.1 to 30 MHz are below 0.1 nT and 0.1 V/m, which indicates low levels of radio frequency contamination.

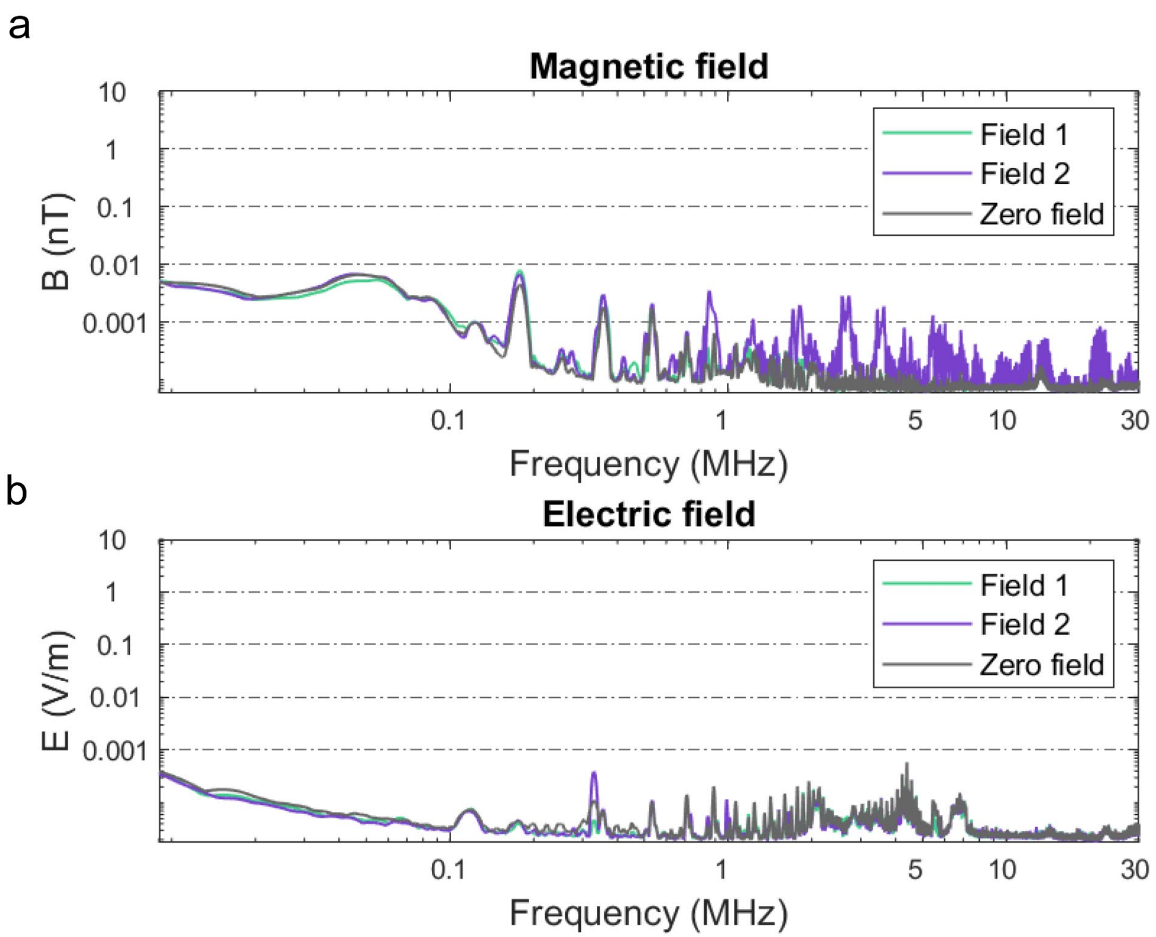

Supplementary Figure 3. Measurements of time-dependent electromagnetic fields. Graphs showing the (a) magnetic and (b) electric components of electromagnetic fields present in the three magnetic conditions within the coil system. measured between 0.1 to 30 MHz are below 0.01 nT and 0.001 V/m, which indicates low levels of radio frequency contamination.
